# Risk factors and survival of patients infected with carbapenem-resistant *Klebsiella pneumoniae* in a KPC endemic setting: a case-control and cohort study

**DOI:** 10.1186/s12879-019-4461-x

**Published:** 2019-10-07

**Authors:** Astrid V. Cienfuegos-Gallet, Ana M. Ocampo de Los Ríos, Patricia Sierra Viana, Faiver Ramirez Brinez, Carlos Restrepo Castro, Gustavo Roncancio Villamil, Helena del Corral Londoño, J. Natalia Jiménez

**Affiliations:** 1 Línea de Epidemiología Molecular Bacteriana, Grupo de Microbiología Básica y Aplicada, Escuela de Microbiología, Universidad de Antioquia, 050010 Medellín, Colombia; 2 IPS Universitaria Clínica León XIII, 050010 Medellín, Colombia; 3 Clínica CardioVID, 050034 Medellín, Colombia

**Keywords:** Case-control study, Cohort study, Carbapenem resistance, *Klebsiella pneumoniae*, length of antibiotic use, innapropriate therapy, risk factors, mortality

## Abstract

**Background:**

Many gaps in the burden of resistant pathogens exist in endemic areas of low- and middle-income economies, especially those endemic for carbapenem resistance. The aim of this study is to evaluate risk factors for carbapenem-resistance, to estimate the association between carbapenem-resistance and all-cause 30-day mortality and to examine whether mortality is mediated by inappropriate therapy.

**Methods:**

A case-control and a cohort study were conducted in one tertiary-care hospital in Medellín, Colombia from 2014 to 2015. Phenotypic and genotypic characterization of isolates was performed. In the case-control study, cases were defined as patients infected with carbapenem-resistant *K. pneumoniae* (CRKP) and controls as patients infected with carbapenem-susceptible *K. pneumoniae* (CSKP). A risk factor analysis was conducted using logistic regression models. In the cohort study, the exposed group was defined as patients infected with CRKP and the non-exposed group as patients infected with CSKP. A survival analysis using an accelerated failure time model with a lognormal distribution was performed to estimate the association between carbapenem resistance and all-cause 30-day-mortality and to examine whether mortality is mediated by inappropriate therapy.

**Results:**

A total of 338 patients were enrolled; 49 were infected with CRKP and 289 with CSKP. Among CRKP isolates CG258 (*n* = 29), ST25 (*n* = 5) and ST307 (*n* = 4) were detected. Of importance, every day of meropenem (OR 1.18, 95%CI 1.10–1.28) and cefepime (OR 1.22, 95%CI 1.03–1.49) use increase the risk of carbapenem resistance. Additional risk factors were previous use of ciprofloxacin (OR 2.37, 95%CI 1.00–5.35) and urinary catheter (OR 2.60, 95%CI 1.25–5.37). Furthermore, a significant lower survival time was estimated for patients infected with CRKP compared to CSKP (Relative Times 0.44, 95%CI 0.24–0.82). The strength of association was reduced when appropriate therapy was included in the model (RT = 0.81 95%CI 0.48–1.37).

**Conclusion:**

Short antibiotic courses had the potential to reduce the selection and transmission of CRKP. A high burden in mortality occurred in patients infected with CRKP in a KPC endemic setting and CRKP leads to increased mortality via inappropriate antibiotic treatment. Furthermore, dissemination of recognized hypervirulent clones could add to the list of challenges for antibiotic resistance control.

## Background

Carbapenem-resistant *Klebsiella pneumoniae* (CRKP) is considered a threat to human health in several regions, including Latin-American countries where high carbapenem-resistance rates have been reported [1]. Strategies for containing carbapenem resistance infections can be greatly improved through the knowledge of context-specific risk factors and understanding hospital transmission dynamics. Exposure to antibiotics has the potential primary role in the risk of CRKP infection, yet the implication of duration of antibiotics in the emergence of antibiotic resistance remains poorly studied. Stronger evidence on the effect of length of antibiotic therapy on antibiotic resistance may prompt the adjustment of treatment courses which are often too long and mostly not evidenced-based [2].

Previous meta-analyses have reported a strong association between carbapenem-resistance and increased risk of death in both crude (OR 3.73; 95% CI 2.02–6.88) and adjusted analysis (OR 2.85; 95% CI 1.88–4.30) [3]. However, whether the increased mortality is due to a higher likelihood of inadequate therapy is still incompletely examined, possible because of limitations on measuring adequate therapy e.g., administration of an active agent, achievement of therapeutic concentration in vivo, time to optimal therapy, optimal route of administration, among others [4].

Most of the risk factors and mortality estimates are from studies in high-income countries and many gaps in the burden of resistant pathogens exist in endemic areas of low- and middle-income economies [5]. Colombia has one of the highest CRKP rates in Latin-America [1] and it is considered endemic for KPC carbapenemases [6]. Colombia’s national surveillance system has reported *K. pneumoniae* as the second most frequent microorganism recovered in both ICU and non-ICU wards (16 and 12%, respectively), with moderate (14%) rates of carbapenem resistance [7]. Although previous studies have described the epidemiology of carbapenem-resistant *K. pneumoniae* infections in Colombia [8], additional information on risk factors for CRKP infection and patient mortality is scarce. To contribute to the understanding of the epidemiology of carbapenem-resistant *K. pneumoniae* infections in a carbapenem resistant endemic country, this study aims primarily to identify risk factors for CRKP infection, including the length of antibiotic use, secondly to estimate the association between carbapenem resistance and all-cause 30-day mortality and to examine whether mortality is mediated by inappropriate therapy in patients with *K. pneumoniae* infections in one tertiary care center in Medellín, Colombia.

## Methods

### Study site and population

This prospective study was conducted from February to March 2014 and from October 2014 to September 2015 in a large (754-beds) university hospital in the city of Medellín-Colombia. Medellín is the second largest city in the country and has a population of 2.5 million inhabitants. The hospital attends children and adult population, with annual discharges of 26,191 in 2014 and 27,869 in 2015. The hospital reported *Klebsiella pneumoniae* infection rates per 1000 hospitalization-days of 0.34 in 2014 and 0.40 in 2015. Hospital meropenem consumption (DDD/100 bed-days) was 10.2 in 2014 and 8 in 2015, and imipenem consumption was 0.4 in 2014 and 0.1 in 2015.

Patients were included the first time *K. pneumoniae* was isolated and inclusion criteria were: (i) adult patients (≥18 years) (ii) from any service and (iii) any type of infection. Patients were excluded if the clinical records or bacterial culture or susceptibility testing results could not be recovered from the microbiology laboratory. After patients were identified from the laboratory records, infectious disease specialists from the study group assessed the infection/colonization status of each patient based on criteria for specific types of infections from CDC/NHSN surveillance definitions [9].

A case-control study was conducted in this tertiary care hospital to identify risk factors for CRKP infection (primary aim) and a cohort study was done to estimate the impact of carbapenem resistance in all-cause 30-day mortality and to examine whether mortality is mediated by inappropriate therapy in patients with *K. pneumoniae* infections (secondary aim).

### Case-control study

A prospective case-control study was conducted to identify risk factors for carbapenem resistance among patients infected by *K. pneumoniae*. Cases were defined as patients infected with carbapenem-resistant *K. pneumoniae* (CRKP) and controls were defined as patients infected with carbapenem-susceptible *K. pneumoniae* (CSKP). All cases and controls that met the inclusion criteria were enrolled in the study. The outcome for the case-control study was resistance to any carbapenem (ertapenem, imipenem, meropenem or doripenem). Information was retrieved from clinical records using a form with standardized definitions. Characterization of study population include: age, sex, type of infection, empirical and targeted antimicrobial therapy, surgical therapy, total length of hospital stay and all-cause mortality. Exposures of interest for the risk factor analysis were: use of antibiotic and duration of antibiotic use in the last 6 months, invasive medical devices at the time or 48 h before sampling, intensive care unit (ICU) hospitalization, healthcare associated infection, surgery in the last year, ICU stay and dialysis in the last 6 months and comorbidities. Because some limitations of a matching design to control for confounding have been described, confounding was controlled in the analytical stage by the use of regression modeling [10, 11]. Confounders included in the analysis were time at risk, defined as the number of days elapsing from admission to sampling, and comorbidities measured as Charlson Index score [12].

### Cohort study

A prospective cohort study was conducted using the previously enrolled population to estimate the association between carbapenem resistance and all-cause 30-day mortality among patients infected by *K. pneumoniae* and to examine whether mortality is mediated by inappropriate therapy. Patients with CRKP infections were considered the exposed group and patients with CSKP infections were considered the non-exposed group. The outcome was all-cause 30-day mortality from the onset of the infection (defined as the day of sampling for bacterial culture). As previously stated, confounding was controlled in the analytical stage by regression modeling. Potential confounders considered in the analysis were: ICU hospitalization at the time of sampling, Charlson index score, transfer from other facility, use of invasive devices at the time of sampling, healthcare-associated infections, surgical treatment (defined as any surgical procedure for source control, including debridement, amputation and surgical drainages) and type of infection. Appropriate therapy was defined as treatment for ≥48 h with at least one antimicrobial agent exhibiting in vitro susceptibility. This was considered as an intermediate variable between carbapenem-resistance and mortality.

### Phenotypic methods for detection of antibiotic resistance

Resistance to carbapenems was defined according to CLSI guidelines [13]. Isolates susceptible to all carbapenems were considered carbapenem-susceptible. Other tested antibiotics included piperacillin/tazobactam, ceftriaxone, ceftazidime, cefepime, amikacin, gentamicin, ciprofloxacin, and tigecycline. Phenotypic testing was done using a VITEK 2 System (bioMérieux, Marcy l’Etoile, France). Clinical breakpoint interpretations were done following CLSI guidelines [13].

### Strain typing of CRKP and CSKP isolates

Variants of *bla*_KPC_ were identified using a molecular beacon-based real-time PCR assay [14] and other carbapenemases - *bla*_VIM_, *bla*_IMP,_ *bla*_NDM_ and *bla*_OXA-48_ - were evaluated using a conventional multiplex PCR [15]. Strain typing was done on all carbapenem-resistant and susceptible *K. pneumoniae* isolates using a molecular beacon based real-time PCR to detect members of the ST258-*tonB79* cluster (ST258, − 512, − 379, − 418, − 554, − 744, − 650, − 683, and − 745) [16]. Isoforms of Tn4410 were detected by PCR [17]. In addition, pulsed-field gel electrophoresis (PFGE) was performed on all CRKP isolates and on a randomly selected subset of CSKP (20%), proportional to the number of isolates collected in each month of the study. Details on the PFGE procedure and analysis were described previously [18]. Representative isolates of each PFGE patterns were further subjected to multi-locus sequence typing (MLST) [19].

### Statistical analysis

A description of the study population was done using absolute and relative frequencies for qualitative variables, and median and interquartile range for quantitative variables with non-normal distribution. To identify risk factors for CRKP infection, bivariate and multivariable analyses were performed using a logistic regression model. The multivariable model was built using purposeful selection of covariables [20]. Briefly, clinical relevant variables and those with *p*-value < 0.25 in the bivariate analysis were selected for inclusion in the model. The model was fitted containing all covariates identified for inclusion at the previous step, and then variables that do not contribute to the model were eliminated. Next, we identified and included back covariables changing > 20% the value of coefficients of the variables retained in the model. The process of refitting, verifying and deleting variables continued until all relevant variables were included in the model. Then the preliminary main effect model was built and quantitative variables remained in the multivariable model after determining the linearity in the logit. The final model was adjusted by time at risk and comorbidities and model estimates were fitted by maximum likelihood with Firth’s correction and profile likelihood confidence intervals to reduce estimate’s bias because of the small number of events per variable [20].

**Fig. 1:**
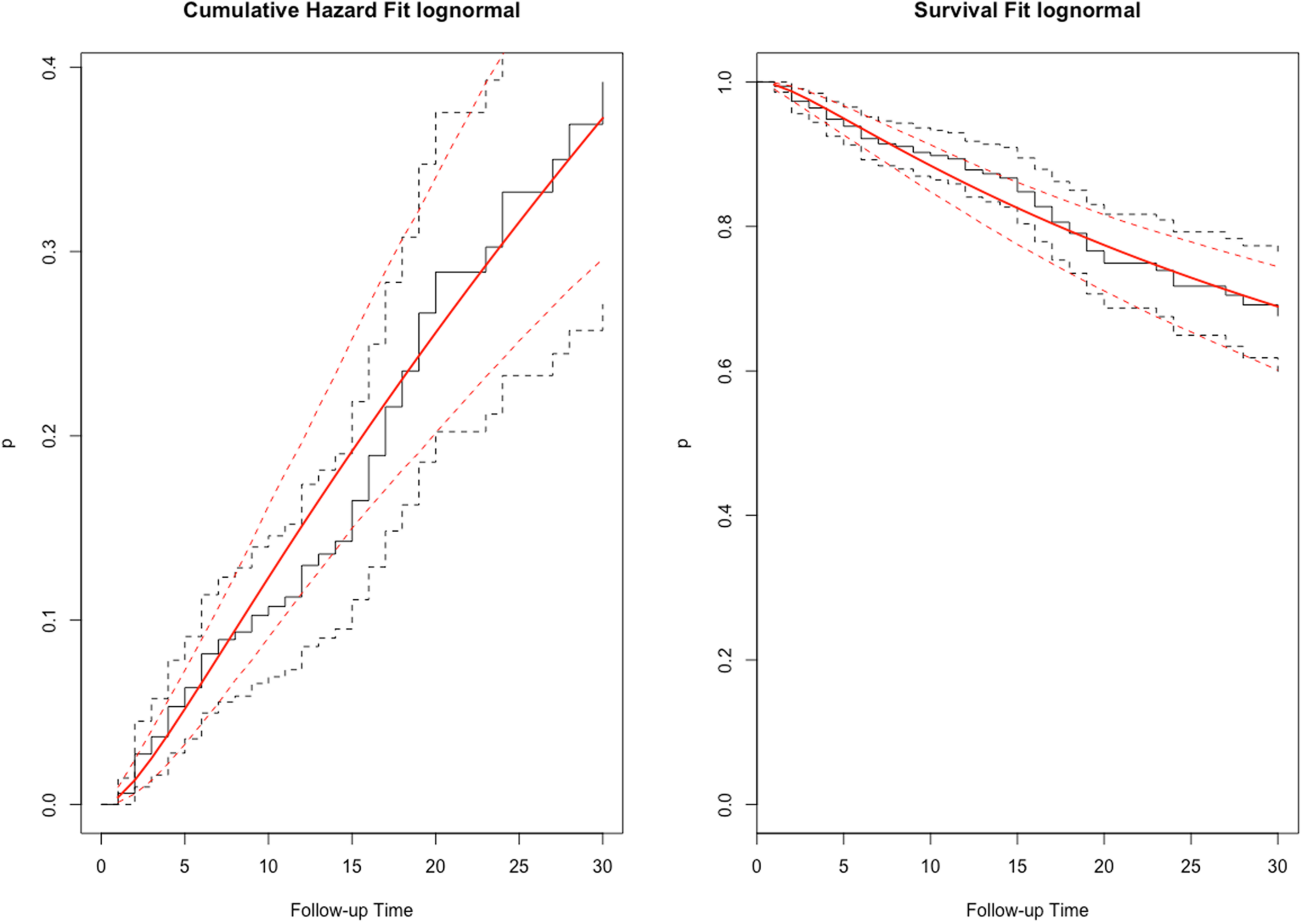
Cumulative hazard and survival functions of patients infected by K. pneumoniae fitted to Lognormal distribution

Descriptive analysis of survival functions using Kaplan-Meier estimates was performed. The Log-rank or the Gehan-Wilcoxon tests was used to evaluate whether selected variables influence the survival function. To estimate the association between carbapenem resistance and all-cause 30-day mortality in *K. pneumoniae* infected patients a survival analysis was done. A parametric survival model was fitted using a Generalized Gamma (GG) distribution because the proportional hazard assumption was not met. The model with Lognormal distribution was selected among GG distributions according the Akaike information Criteria (AIC). The bivariate and final multivariable accelerated failure time (AFT) models were fitted using this distribution (Fig. 1). This analysis estimates the ‘Relative Times’. As proposed by Cox [21], given an unexposed population and an exposed population with survival function of S_0_(t) and S_1_(t), respectively, the relative times are defined for 0 < *p* < 1 as the ratio of the corresponding quantile functions, RT*(p)* = t_1_*(p)*/t_0_*(p)* = S_1_^− 1^(1-*p*)/S_0_^− 1^(1-*p*), where S_1_^− 1^ and S_0_^− 1^, are the inverses of the survival function. Then, the time required for any percent of individuals in the exposed population to experience the event of interest is RT-fold the time for the same proportion of events to occur in the non-exposed population [21]. To examine whether appropriate therapy mediates the effect of carbapenem resistance on mortality, this variable was included in the AFT final model. All statistical analyses were implemented in RStudio Version 1.0.136 [22].

## Results

### Clinical characteristics of patients

A total 338 patients infected by *K. pneumoniae* were enrolled in the study, after excluding three patients with no isolate recovered for microbiological testing. Forty-nine (14.50%) patients were infected by CRKP and 289 (85.50%) were infected by CSKP. Most patients were male (*n* = 198, 58.58%) and older (median age 67 years, IQR 51–77). The most frequent infection was urinary tract infection (UTI) (*n* = 95, 28.11%), followed by intra-abdominal infection (*n* = 73, 21.60%). The median of total length of hospital stay from admission to discharge was 19 days (IQR 10–34) with a maximum stay of 194 days (Table 1). Overall, 15.09% (*n* = 51) of patients were in ICU at the onset of infection, 60.36% (*n* = 204) had previous hospitalization, 67.75% (*n* = 229) had previous use of antibiotics, mainly piperacillin/tazobactam (*n* = 107, 31.66%), and 95.86% (*n* = 324) had at least one comorbid illness. The median of Charlson index score was 4 (IQR 2–6) (Table 2).

**Table 1:** Characteristics of *K. pneumoniae* infections according susceptibility to carbapenems

Characteristics	Carbapenem-susceptible *K. pneumoniae n* = 289		Carbapenem-resistant *K. pneumoniae n* = 49		Total *K. pneumoniae* infections *n* = 338	
	n	%	n	%	n	%
Site of infection						
Urinary tract infection (UTI)	82	28.37	13	26.53	95	28.11
Intra-abdominal infection	67	23.18	6	12.24	73	21.60
Surgical site infection	24	8.30	8	16.33	32	9.47
Bacteriemia	25	8.65	5	10.20	30	8.88
Catheter associated UTI	19	6.57	6	12.24	25	7.40
Pneumonia	19	6.57	3	6.12	22	6.51
Skin and soft tissue infections	18	6.23	2	4.08	20	5.92
Empirical treatment	262	90.66	45	91.84	307	90.83
Piperacillin/Tazobactam	169	58.48	21	42.86	190	56.21
Meropenem	59	20.42	21	42.86	80	23.67
Targeted treatment	270	93.43	39	79.59	309	91.42
Meropenem	125	43.25	30	61.22	155	45.86
Ciprofloxacin	49	16.96	4	8.16	53	15.68
Tigecycline	2	0.69	18	36.73	20	5.92
Colistin	0	0.00	10	20.41	10	2.96
Combined therapy	45	15.57	33	67.35	78	23.08
Surgical treatment	89	30.80	11	22.45	100	29.59
Hospital length stay (median, IQR)	18	(10–33)	26	(13–42)	19	(10–34)
30-day mortality (all-causes)	46	15.92	16	32.65	62	18.34

**Table 2:** Bivariate analysis of risk factors for carbapenem-resistant *K. pneumoniae* infection in patients infected with *K. pneumoniae*

Variables	Carbapenem-susceptible *K. pneumoniae n* = 289		Carbapenem-resistant *K. pneumoniae n* = 49		Total *K. pneumoniae* infections *n* = 338		OR (95% CI)
	n	%	n	%	n	%	
Sociodemographics							
Age in years (median, IQR)	67	(51–77)	64	(55–74)	67	(51–76)	0.99 (0.97–1.01)
Male sex	164	56.75	34	69.39	198	58.58	1.73 (0.90–3.31)
Transfer from another facility	111	38.41	18	36.73	129	38.17	0.93 (0.50–1.74)
Clinical characteristics							
Time at risk in days (median, IQR)	3	(1–12)	10	(1–26)	3	(1–14)	**1.03 (1.01–1.05)**
ICU hospitalization	38	13.15	13	26.53	51	15.09	**2.38 (1.16–4.90)**
Healthcare associated infection	118	40.83	30	61.22	148	43.79	**2.28 (1.23–4.25)**
Invasive devices	143	49.48	38	77.55	181	53.55	**3.5 (1.73–7.17)**
Urinary catheter	69	23.88	21	42.86	90	26.63	**2.39 (1.28–4.47)**
Central venous catheter	53	18.34	21	42.86	74	21.89	**3.33 (1.77–6.32)**
Enteral nutrition	30	10.38	11	22.45	41	12.13	**2.49 (1.16–5.39)**
Mechanical ventilation	25	8.65	11	22.45	36	10.65	**3.05 (1.40–6.71)**
Medical history (previous year)							
Surgery	105	36.33	23	46.94	128	37.87	1.55 (0.84–2.85)
Medical history (previous 6 months)							
Hospitalization	171	59.17	33	67.35	204	60.36	1.42 (0.75–2.70)
ICU stay	31	10.73	8	16.33	39	11.54	1.62 (0.70–3.77)
Dialysis	37	12.80	11	22.45	48	14.20	1.97 (0.93–4.19)
Previous use of antibiotics	186	64.36	43	87.76	229	67.75	**2.59 (1.26–5.31)**
Piperacilin/Tazobactam	90	31.14	17	34.69	107	31.66	1.17 (0.62–2.22)
Aztreonam	5	1.73	2	4.08	7	2.07	2.41 (0.45–12.82)
Cefepime	4	1.38	7	14.29	11	3.25	**11.87 (3.33–42.30)**
Meropenem	22	7.61	20	40.81	42	12.43	**8.36 (4.08–17.13)**
Ciprofloxacin	36	12.46	14	28.57	50	14.79	**2.81 (1.38–5.72)**
Amikacina	9	3.11	6	12.24	15	4.44	**4.34 (1.47–12.80)**
Previous days of antibiotic use(number of days) (median, IQR)							
Piperacillin/Tazobactam	7	4–10	6.5	4–9.5	7	4–10	1.17 (0.62–2.22)
Aztreonam	4	3–6	8.5	8–9	6	3–8	1.25 (0.97–1.61)
Cefepime	7.5	4–11.5	8	7–12	8	7–12	**1.27 (1.09–1.49)**
Meropenem	9	5–12	13.5	8–18.5	11	6–14	**1.20 (1.13–1.28)**
Ciprofloxacin	6	4–16	7	2.5–10	6.5	4–13	1.03 (0.97–1.10)
Amikacina	4	1–7	9.5	7–20	7	1–12	**1.18 (1.04–1.33)**
Comorbidities	276	95.50	48	97.96	324	95.86	
Charlson Index (median, IQR)	4	(2–6)	4	(3–5)	4	(2–6)	1.002 (0.88–1.13)
Cardiovascular disease	137	47.40	23	46.94	160	47.34	0.98 (0.53–1.80)
Diabetes Mellitus	91	31.49	12	24.49	103	30.47	0.71 (0.35–1.41)
Cancer	52	17.99	10	20.41	62	18.34	1.17 (0.55–2.49)
Renal chronic disease	53	18.34	5	10.20	58	17.16	0.51 (0.19–1.34)
COPD	50	17.30	7	14.29	57	16.86	0.80 (0.34–1.87)

**Significant at α < 0.05**

*ICU* Intensive Care Unit, *COPD* Chronic Obstructive Pulmonary Disease

^‡^Included in the multivariable analysis (*p* < 0.25)

### Isolates susceptibility testing and genotyping

A higher proportion of CRKP versus CSKP had MICs in the resistant range to other antibiotic classes: 59.18% vs 0.35% to amikacin, 53.06% vs 24.91% to gentamicin, 79.59% vs 31.83% to ciprofloxacin, 69.05% vs 30.50% to tigecycline and 27.27% vs 0.41% to colistin. Multidrug resistance, defined as resistance to at least three antibiotic classes, was common in CRKP (*n* = 40, 83.33%) compared to CSKP (*n* = 41, 14.19%); however, most MDR isolates were susceptible to colistin (81.16%). The most frequent profiles in CRKP isolates were resistance to ertapenem + imipenem + meropenem + amikacin + gentamicin + ciprofloxacin + tigecycline + colistin (*n* = 9, 18.3%) and ertapenem + imipenem + meropenem + amikacin + ciprofloxacin + tigecycline (*n* = 8, 16.3%). Although CSKP isolates were mostly multi-susceptible (*n* = 135, 46.7%), the second most common resistance profile was MDR with resistance to tigecycline + ciprofloxacin + gentamicin (*n* = 30, 10.4%).

**Fig. 2:**
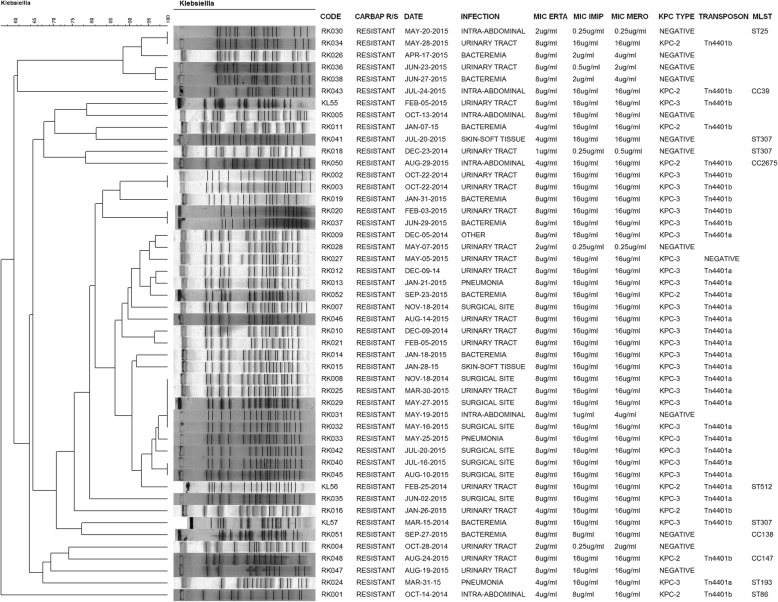
PFGE dendrogram showing the genetic relationship among 49 isolates of carbapenem-resistant *K. pnuemoniae*. The Dice similarity coefficient and the unweighted pair group method with arithmetic averages were used for dendogram generation in Bionumerics software. The cluster of isolates belonging to CG258 had > 80% genetic relatedness

**Fig. 3:**
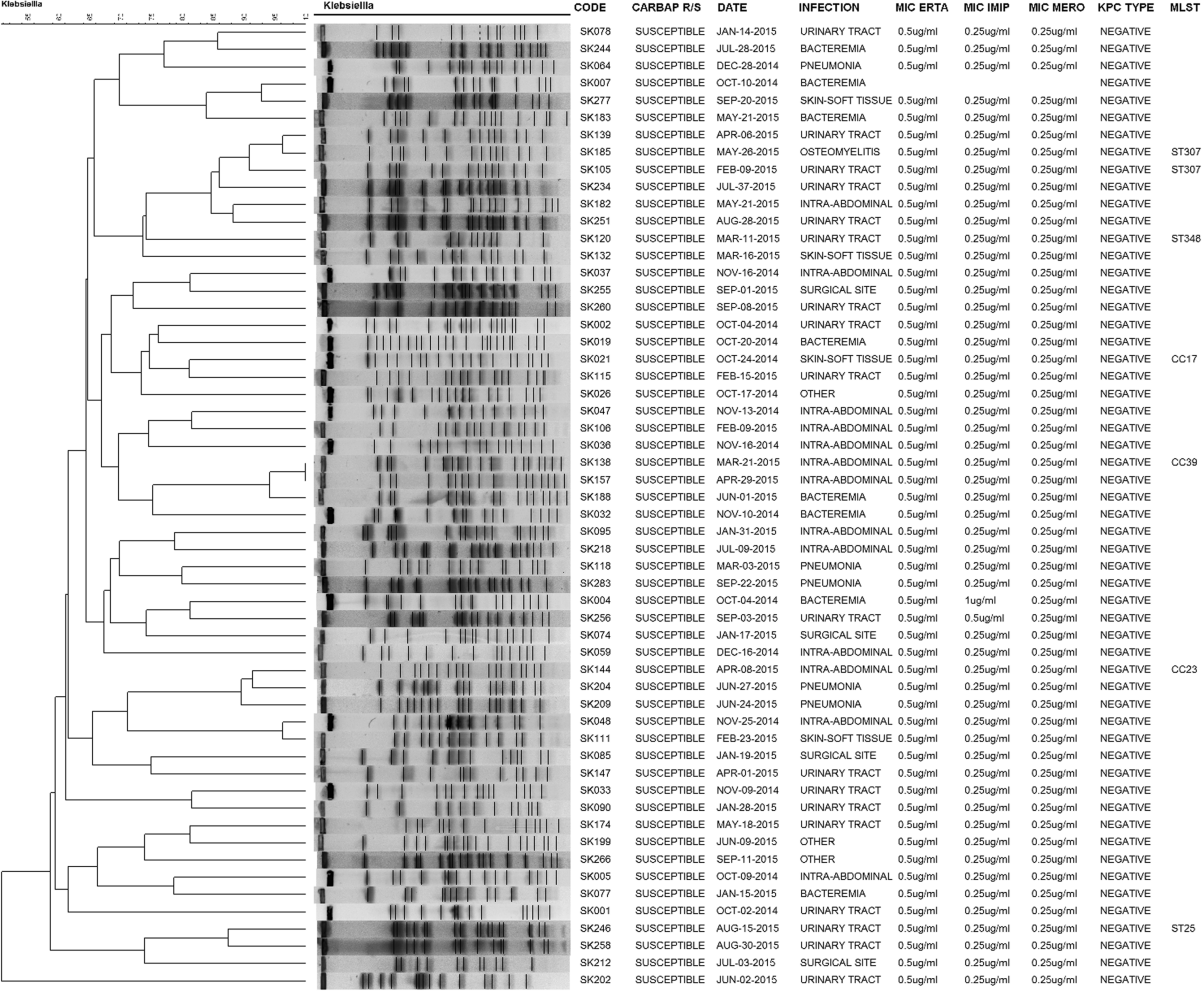
PFGE dendrogram showing the genetic relationship among 56 isolates of carbapenem-susceptible *K. pneumoniae*. The Dice similarity coefficient and the unweighted pair group method with arithmetic averages were used for dendogram generation in Bionumerics software. Most isolates were not related genetically according to the Dice similarity coefficient < 80%

Carbapenemases genes detected in CRKP were KPC-3 (*n* = 28, 57.14%) and KPC-2 (*n* = 9, 18.37%). Other carbapenemases genes were not detected. Most CRKP were CG258 (*n* = 28, 57.14%), and one isolate within this cluster proved to be ST512. Other clones were detected including ST25 (*n* = 5), ST307 (*n* = 4), and one isolate each of ST193, ST86, CC138, CC147, CC2675 and CC39 and the new STs ST2833, ST2834, and ST2835. Among CSKP ST307 (*n* = 6) and ST25 (*n* = 3) were also detected, in addition to the new ST2836 (*n* = 1) and ST2837 (*n* = 1). PFGE typing revealed a large cluster of CRKP isolates belonging to the CG258 and clonal diversity among CSKP isolates (Figs. 2 and 3, respectively).

### Risk factors for carbapemen-resistant *K. pneumoniae* infection

The characteristics of patients infected by CRKP and CSKP are presented in Table 2. The time at risk was longer in CRKP (median 10 days, IQR 1–26) compared to CSKP (median 3 days, IQR 1–12). CRKP patients had higher ICU hospitalization (*n* = 51, 26.53%), invasive devices at the time or 48 h before sampling (*n* = 38, 77.55%) and previous exposure to antibiotics (*n* = 43, 87.76%) compared to CSKP patients (Table 2). Bivariate logistic regression analysis showed several of these characteristics were associated to CRKP infection, including time at risk, ICU hospitalization, use of invasive devices (urinary catheter, central venous catheter, enteral nutrition and mechanical ventilation), and previous use of antibiotics, mainly meropenem and ciprofloxacin (Table 2). No previous exposure to imipenem was recorded in any of the patients included; only two patients had previous exposure to ertapenem (one CRKP and one CSKP infected patients) and one CRKP patient had exposure to doripenem.

After adjustment for time at risk and comorbidities to control for confounding, the final multivariable model showed that previous days of meropenem use (OR 1.18, 95%CI 1.10–1.28), previous days of cefepime use (OR 1.22, 95%CI 1.03–1.49), previous use of ciprofloxacin (OR 2.37, 95%CI 1.00–5.35) and urinary catheter (2.60, 95%CI 1.25–5.37) were risk factors for CRKP infection (Table 3).

**Table 3:** Multivariable logistic regression analyses of risk factors for carbapenem resistance among *K. pneumoniae* infected patients adjusted for time at risk and comobordities

Risk factors	OR (95% CI)
Previous use of meropenem (days)	1.18 (1.10–1.28)
Previous use of cefepime (days)	1.22 (1.03–1.49)
Previous use of ciprofloxacin	2.37 (1.00–5.35)
Urinary catheter	2.60 (1.25–5.37)

### Carbapenem resistance, inappropriate therapy and all-cause 30-day mortality in patients infected by *K. pneumoniae*

All-cause 30-day mortality among patients infected by *K. pneumoniae* was 18.34% (*n* = 62) and median time to death after the onset of infection was 8.5 days (IQR 4–17).

**Fig. 4:**
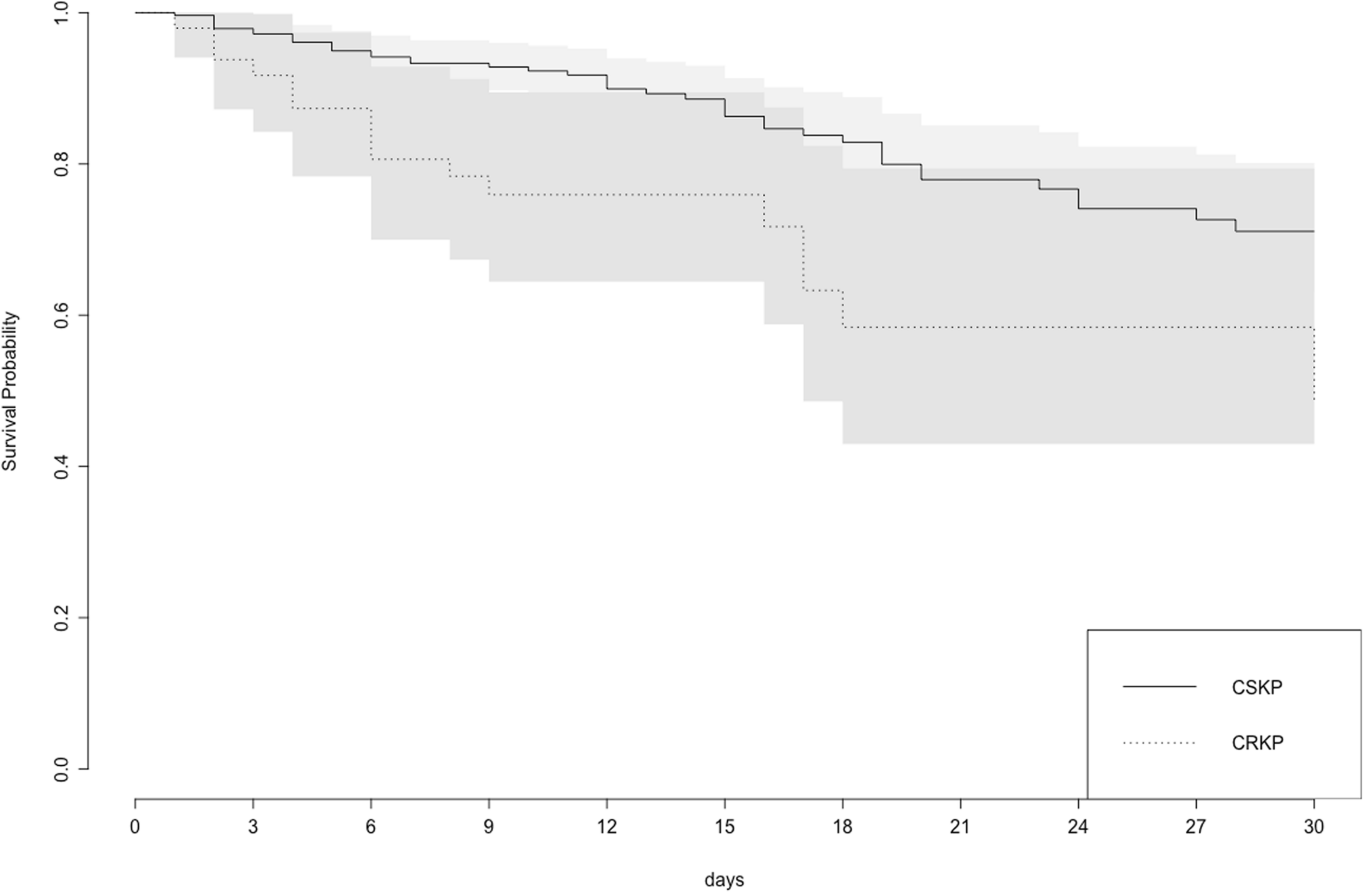
Kaplan-Meier survival functions and its 95% confidence intervals of patients infected by *K. pneumoniae* according to carbapenem resistance. Patients infected with CRKP showed significantly lower survival probability than patients infected with CSKP (Log-rank test, *p* = 0.0032). CSKP: carbapenem-susceptible *K. pneumoniae*. CRKP: carbapenem-resistant *K. pneumoniae*

CRKP infected patients had significantly higher crude 30-day mortality compared to CSKP (32.65% vs. 15.92%); however, the median number of days until death was similar for both groups (CRKP 6 days IQR 3.5–16.5 vs. CSKP 10.5 days IQR 4–17, *p* = 0,556). Among CRKP patients, all-cause 30-day mortality in KPC-producing infected patients was 35.13% (13/37) compared to 25.00% (3/12) in the non-KPC-producing infected group. Kaplan-Meier survival functions showed CRKP infected patients had significantly lower survival probability than CSKP infected patients (Log-rank test, *p* = 0.0032) (Fig. 4).

In the AFT multivariable model, CRKP was a strong risk factor for mortality (RT 0.44 95%CI 0.24–0.82) after adjustment for age, healthcare associated infection, ICU hospitalization, comorbidities (Charlson Index score) and type of infection. CRKP infection reduced the survival time of patients by approximately 60% compared to the CSKP group adjusted for other variables in the model (Table 4). Finally, to examine whether increased mortality occurs via inappropriate therapy, the intermediate variable (appropriate therapy) was included in the model, which resulted in an increase of the survival time in carbapenem-resistant infected patients (RT = 0.81 95%CI 0.48–1.37).

**Table 4:** Bivariate and multivariable Accelerated Failure Time model estimates of the survival time within 30 days after the onset of infection among patients infected with *K. pneumoniae*

Variables	Survivors (*n* = 276)		Non-survivors (*n* = 62)		Relative times (95% CI)	Adjusted relative times (95% CI)
	n	%	n	%		
CRKP infection	33	11.96	16	25.81	0.42 (0.22–0.76)	0.44 (0.24–0.84)
Male sex	161	58.33	37	59.68	0.91 (0.55–1.49)	–
Age in years (median, IQR)	65	(51–76)	71	(55–78)	0.99 (0.97–1.00)	0.99 (0.98–1.01)
Transfer from another facility	103	37.32	26	41.94	1.21 (0.74–1.96)	–
ICU hospitalization	31	11.23	20	32.26	0.61 (0.33–1.13)	0.77 (0.41–1.45)
Mechanical ventilation	23	8.33	13	20.97	0.92 (0.46–1.83)	–
Charlson Index score (median, IQR)	4	(2–6)	4	(3–6)	0.92 (0.84–1.01)	0.90 (0.78–1.03)
Healthcare associated infection	103	41.53	33	58.93	0.58 (0.34–0.98)	0.72 (0.40–1.30)
Surgical treatment	81	29.35	19	30.65	1.22 (0.73–2.05)	–
Type of infection						
UTI-CAUTI	109	39.49	11	17.74	reference	
Pneumonia	17	6.16	7	11.29	0.58 (0.22–1.48)	0.54 (0.20–1.44)
Bloodstream infection	32	11.59	16	25.81	0.52 (0.24–1.11)	0.58 (0.24–1.38)
Surgical site infection	24	8.70	8	12.90	0.62 (0.25–1.54)	0.87 (0.32–2.40)
Intra-abdominal	56	20.29	17	27.42	0.53 (0.26–1.05)	0.45 (0.21–0.94)
Other	38	13.77	3	4.84	2.44 (0.88–6.76)	1.93 (0.68–5.46)

## Discussion

Our results highlighted the role of the length of previous therapy with meropenem and cefepime in the emergence of carbapenem resistance, as well as the role of previous use of ciprofloxacin and urinary catheters are important risk factors for CRKP infection in a KPC endemic setting. In addition, a significant reduction of survival time after confounding adjustment was found in patients infected by CRKP compared to CSKP. Furthermore, genotyping of *K. pneumoniae* isolates also revealed that CG258 still continue to cause most of CRKP infections in our hospital, and notably the recently emerging carbapenem-resistant and hypervirulent ST307 clones were found among CRKP and CSKP infecting isolates.

Of note, in our study a detailed characterization of antibiotic exposure (i.e., days of previous antibiotic therapy) showed that length of meropenem and cefepime use increases the odds of CRKP infection among the exposed group (dose- response effect). The odds of infection with CRKP increase by 19% for every day of meropenem use and 22% for every day of cefepime use. These results suggest the potential protective effect of short-course therapy to minimize the impact of antibiotic use on the emergence of drug-resistant bacteria.

Consistent with our results, previous studies have found exposure to carbapenems [23, 24], ciprofloxacin [25–27], extended spectrum cephalosporins [27], and days of antibiotic use [28, 29], were risk factors for CRKP infection. Broad-spectrum antibiotics, such as cefepime and ciprofloxacin, probably select for colonization or infection with more resistant bacteria, not only CRKP but also MDR [30]. In addition, exposure to ciprofloxacin or extended spectrum cephalosporins probably co-selects carbapenem-resistant strains because *qnr* genes responsible for low-level ciprofloxacin resistance, *bla*_CTX-M_ a gene coding for an extended spectrum betalactamase, and *bla*_KPC_ genes might be found in the same mobile elements within the same strain [31]. Alternatively, *bla*_KPC_-harboring plasmids can be transferred into ciprofloxacin-resistant strains selected previously.

Of importance, a previous study showed an interaction effect between the previous use of carbapenem and fluoroquinolones, revealing that longer exposures to both antibiotics boosted the risk of CRKP infection (OR 1.02, 95%CI 1.00–1.04) [29]. These results underscore the importance of ongoing antimicrobial stewardship programs in our hospital to optimize the treatment of infections and reduce antibiotic misuse leading to the selection of antibiotic resistant bacteria.

An additional recognized risk factor for CRKP infection/colonization is the use of medical invasive devices such as urinary catheter [32], mechanical ventilation [28] and venous catheterization [33]. Of note, our results showed that patients using urinary catheters had a 2.60-fold odd of infection with CRKP compared to patients without urinary catheters. This suggests that strategies for preventing infections related to the use of urinary catheter, such as appropriate indication and length of use, training of persons performing the procedure and practices of hand hygiene and standard precautions, are useful to reduce infections with CRKP in our setting. Several studies have shown that ICU stay is also associated to CRKP infection/colonization [26, 34]. However, our results showed only 26.53% of the patients were hospitalized in ICUs at the time of sampling, revealing that most CRKP infections occurred in general wards. Although in our study, endoscopy procedures were not evaluated, these procedures have been also associated to CRKP transmission in both endemic and epidemic setting. In fact, endoscopy was associated to carbapenem resistance in different *K. pneumoniae* strains, including KPC producing (OR 6.71, 95%CI 1.25–36.0) and porin-ertapenem resistant- *K. pneumoniae* (6.12, 95%CI 1.46–25.6) in patients with healthcare infections [35].

Significantly, in this study a higher all-cause 30-day mortality was found in patients infected with CRKP than in those infected with CSKP. Estimates of mortality due to CRKP from several studies have shown conflicting results. After adjustment for severity of illness, comorbidities, ICU stay and/or use of invasive devices, some studies have reported CRKP did not contribute to the mortality of infected patients [25, 33, 36], while others have reported a significantly high all-cause mortality in the CRKP group [23, 27].

A previous meta-analysis [37] reported an overall pooled mortality of 42.14% (95%CI 37.06–47.31) in CRKP and 21.16% (95%CI 16.07–26.79) in CSKP group. A second meta-analyses in patients with bacteremia reported a higher crude mortality among CRKP-infected patients compared to CSKP-infected patients (unadjusted OR 2.16, 95%CI 1.78–2.62) [38], with high mortality observed among studies including primarily KPC-producing strains among CRKP (OR 2.92, 95%CI 2.15–3.95) [38]. In our study, the overall all-cause mortality was also higher in CRKP infected patients (32.65% vs 17.30% in CSKP group) and 35% patients died in the KPC-*K. pneumoniae* infected group. Most worryingly, the present study showed an approximately 60% reduction in the survival time of CRKP infected patients (RT 0.44 95%CI 0.24–0.82) after adjustment for age, healthcare associated infection, ICU hospitalization, comorbidities (Charlson Index score) and type of infection.

Several studies have underscored the importance of appropriate antibiotic treatment in the survival of CRKP-infected patients. In the previous meta-analysis the OR for mortality of CRKP compared to CSKP in the subgroup of patients receiving appropriate initial treatment was reduced from 2.66 (95%CI 1.83–3.87) to 2.21 (95%CI 1.29–3.81) [38]. In our study, the strength of the association between carbapenem resistance and mortality was reduced when appropriate therapy was included in the model (RT = 0.81 95%CI 0.48–1.37). These results support that inappropriate therapy is an intermediate variable between the exposure (infection with a resistant bacteria) and the outcome (mortality), and consequently CRKP leads to increased mortality via inappropriate antibiotic treatment [39]. In this study, inappropriate therapy was defined as the use of antibiotics with no in vitro activity against the identified microorganisms as adopted by the American Thoracic Society and the Infectious Diseases Society of America [40, 41]. It is also necessary to recognize that a more useful measure would be the adequacy of therapy, which requires not only the selection of the correct (appropriate) antibiotic but also the optimal dose and the proper route of administration to ensure penetration to the site of infection. However, this information is difficult to obtain in observational studies.

The carbapenem-resistant *K. pneumoniae* pandemic is driven by the global dissemination of CC258 [6], whose members include ST258, ST512, ST11 and ST340. Among these clones, ST512 and ST258 have been frequently reported in CRKP isolates from Colombia [8]. In our hospital CG258 is the main CRKP circulating clone which contrasts to previous reports in other hospitals in Medellin [8]. A previous work in the same center revealed annual fluctuations of CG258 among CRKP isolates: 83% in 2012, 66% in 2013, 76% in 2014 and 75% in 2015, with approximately 20 cases/year, suggesting that most of the carbapenem-resistant infections are caused by the dissemination of the successful CG258 clone. ST307 isolates had been detected in our hospital since 2013 [42]; however, CG258 carbapenem-resistant isolates still predominated in our setting. It has been suggested that ST307 is a virulent clone that could possibly replace ST258 in Italy, leading to a multi-clonal epidemic [43]. In addition, ST25 were detected for the first time in our hospital in 2015. This clone has been related to a hypermucoviscous hypervirulent phenotype associated with severe infections [44]. These findings indicate CRKP endemics may be worsened by the dissemination of simultaneously carbapenem-resistant and hypervirulent clones.

In our hospital several infection control measures targeting resistant organisms are in place including protocols, training and signaling for precaution of pathogen transmission, active surveillance of the most common antimicrobial resistant pathogens (including CRKP), verification of adherence to precaution measures, cohorts of colonized/infected patients in internal medicine, hematology, nephrology, surgery and ICU units, active search of ESBL and carbapenemas-producing bacteria and computer system alerts for bacterial species displaying a carbapenemase profile.

This study has some limitations. First, there is a lack of information about the colonization status of the majority of patients because surveillance cultures are not implemented in all patients. In our study, colonization data was available for less than half of the study population (46.75%). It is possible that the colonization status and colonization pressure could be additional risk factors for infection with resistant bacteria [28], considering that molecular typing also revealed the predominance of the CG258 among CRKP infected patient. Second, available information about antibiotic exposure was limited to hospital registries, consequently outpatient antibiotic therapy was not retrieved and it can affect results from this study. Similarly, information on length of previous hospital stay was not collected, and it may confound the association between antibiotic use and carbapenem resistance, since longer length of previous antibiotic therapy may reflect a longer previous length of hospital stay, which can increase the risk of acquiring a drug-resistant bacteria. Additionally, because of missing data on susceptibility to polymixin B and fosfomycin, analysis of appropriateness of treatment was not performed in all patients (316/338 patients had complete information to define appropriate therapy). In addition, all-cause, but not attributable mortality was assessed in this population. Finally, results obtained here from an institution in an endemic region for carbapenem resistance could not be generalized to other institutions with low prevalence of carbapenem resistance. It is also important to consider that comparisons were done between CRKP vs. CSKP infected patients, and consequently factors identified here are those involved in the emergence of carbapenem resistance [45]. Likewise, additional risk factors might be identified if uninfected patients are used as control group.

## Conclusions

The risk factors for CRKP infection were days of meropenem and cefepime exposure and use of urinary catheters. These findings support the need to continue strict policies for antibiotic use in hospitals and careful management of patients with medical devices, mainly urinary catheters. In this study it was also observed that CRKP infection was associated with higher mortality and that CRKP leads to increased mortality via inappropriate antibiotic treatment. These results demonstrated the threat of antibiotic resistance on patient survival in endemic settings. In addition, the detection of recognized carbapenem-resistant and hypervirulent clones alerts the future challenges to be faced if efforts are not maintained to control antibiotic resistance emergence and dissemination.

## Data Availability

The datasets used and/or analyzed during the current study are available from the corresponding author on reasonable request.

## Abbreviations

AFT: Accelerated failure time model
AIC: Akaike information Criteria
CAUTI: Catheter associated urinary tract infection
CC: Clonal complex
CDC/NHSN: Center for Disease Control and Prevention’s National Healthcare Safety Network
CG: Clonal group
CI: Confidence interval
CLSI: Clinical Laboratory Standards Institute
COPD: Chronic Obstructive Pulmonary Disease
CRKP: Carbapenem-resistant *K. pneumoniae*
CSKP: Carbapenem-susceptible *K. pneumoniae*
DDD: Defined daily doses
ESBL: Extended-spectrum beta-lactamase
GG: Generalized gamma
ICU: Intensive care unit
IQR: Interquartile range
KPC: *K. pneumoniae* carbapenemase
MDR: Multidrug resistant
MIC: Minimum inhibitory concentration
MLST: Multi-locus sequence typing
OR: Odds ratio
PCR: Polymerase chain reaction
PFGE: Pulsed-field gel electrophoresis
RT: Relative times
ST: Sequence type
UTI: Urinary tract infection
